# Ecological biomechanics of damage to macroalgae

**DOI:** 10.3389/fpls.2022.981904

**Published:** 2022-08-25

**Authors:** Nicholas P. Burnett, M. A. R. Koehl

**Affiliations:** ^1^Department of Neurobiology, Physiology, and Behavior, University of California, Davis, Davis, CA, United States; ^2^Department of Integrative Biology, University of California, Berkeley, Berkeley, CA, United States

**Keywords:** hydrodynamics, material properties, strength, breakage, wounds, herbivory, drag

## Abstract

Macroalgae provide food and habitat to a diversity of organisms in marine systems, so structural damage and breakage of thallus tissue can have important ecological consequences for the composition and dynamics of marine communities. Common sources of macroalgal damage include breakage by hydrodynamic forces imposed by ambient water currents and waves, tissue consumption by herbivores, and injuries due to epibionts. Many macroalgal species have biomechanical designs that minimize damage by these sources, such as flexibly reconfiguring into streamlined shapes in flow, having either strong or extensible tissues that are tough, and having chemical and morphological defenses against herbivores and epibionts. If damage occurs, some macroalgae have tissue properties that prevent cracks from propagating or that facilitate tissue breakage in certain places, allowing the remainder of the thallus to survive. In contrast to these mechanisms of damage control, some macroalgae use breakage to aid dispersal, while others simply complete their reproduction prior to seasonally-predictable periods of damage (e.g., storm seasons). Once damage occurs, macroalgae have a variety of biomechanical responses, including increasing tissue strength, thickening support structures, or altering thallus shape. Thus, macroalgae have myriad biomechanical strategies for preventing, controlling, and responding to structural damage that can occur throughout their lives.

## Introduction

Macroalgae play critical roles in marine ecosystems (Steneck et al., 2002; Schiel and Foster, 2015), so damage that alters their size or morphology can have serious ecological consequences. For example, large macroalgae provide more habitat space and resources for the diverse organisms that live on or amongst their fronds than do small seaweeds (Steneck et al., 2002; Graham et al., 2007; Christie et al., 2009). Large, highly-branched macroalgae also shape surrounding benthic communities by intercepting light, changing sedimentation patterns, and scouring nearby organisms off the substratum (Kennelly, 1989; Arkema et al., 2009; Hughes, 2010). Furthermore, aggregations of macroalgae alter ambient water flow (slowing currents, attenuating waves, altering turbulence spectra), thereby protecting organisms and shorelines from hydrodynamic damage (Denny, 2021; Zhu et al., 2021b; Koehl, 2022).

The ecological effects of damage to macroalgae depend on which species are injured, which parts of their thalli are harmed, and the scale of the damage. For instance, herbivores may eat only certain species (e.g., Toth and Pavia, 2002), life stages (e.g., Van Alstyne et al., 2001; Chenelot and Konar, 2007), or specific macroalgal structures (e.g., Fralick et al., 1974), while seasonal storms rip away some species and sizes of macroalgae more than others (Black, 1976; Koehl, 1999, 2022). Minor damage to macroalgae diminishes provision of food and habitat, whereas major damage disrupts community structure (Johnson and Mann, 1986; Chenelot and Konar, 2007; Poore et al., 2014). However, periodic breakage of competitively-dominant macroalgae enhances local biodiversity (Sousa, 1979). Furthermore, broken macroalgae become organic detritus that enriches benthic communities (Duggins and Eckman, 1997; Krumhansl and Scheibling, 2012; de Bettignies et al., 2013b).

We consider damage to macroalgae through the lens of ecological biomechanics. Biomechanics is the study of how biological structures perform mechanical functions. The integration of biomechanics and ecology (“ecological biomechanics,” Koehl, 1999; “ecomechanics,” Denny, 2012; Higham et al., 2021; “mechanical ecology,” Bauer et al., 2020) provides an ideal framework to study macroalgal damage that incorporates sources of injury in the environment, structural design and tissue material properties that resist or compensate for damage, and effects of morphological changes caused by breakage on the performance of the macroalgae in natural habitats, and thus on their survival and reproduction.

## Sources of damage

### Hydrodynamic forces

Macroalgae encounter currents and waves. This water motion benefits macroalgae by delivering nutrients, removing wastes, and dispersing gametes and spores (Norton et al., 1981; Koehl, 1984, 1999; Denny, 1988; Vogel, 1996; Hurd, 2000). However, moving water also exerts hydrodynamic forces (drag and acceleration reaction) on macroalgae that can damage or dislodge them. Drag is proportional to the square of water velocity relative to a macroalga, its planform area, and the shape it takes in the flow, while acceleration reaction depends on water acceleration relative to a macroalga, its volume and shape (details in Koehl, 1976; Denny et al., 1985; Vogel, 1996). Hydrodynamic forces on macroalgae vary over different time scales (seconds in a wave; hours over a tidal cycle; months as seasonal storm patterns change; Seymour et al., 1989; Gaylord, 1999; Koehl, 2022) and spatial scales (centimeters to meters of substratum rugosity and neighboring organisms; kilometers of coastal topography and orientation; O’Donnell and Denny, 2008; Nickols et al., 2012).

**Figure 1:**
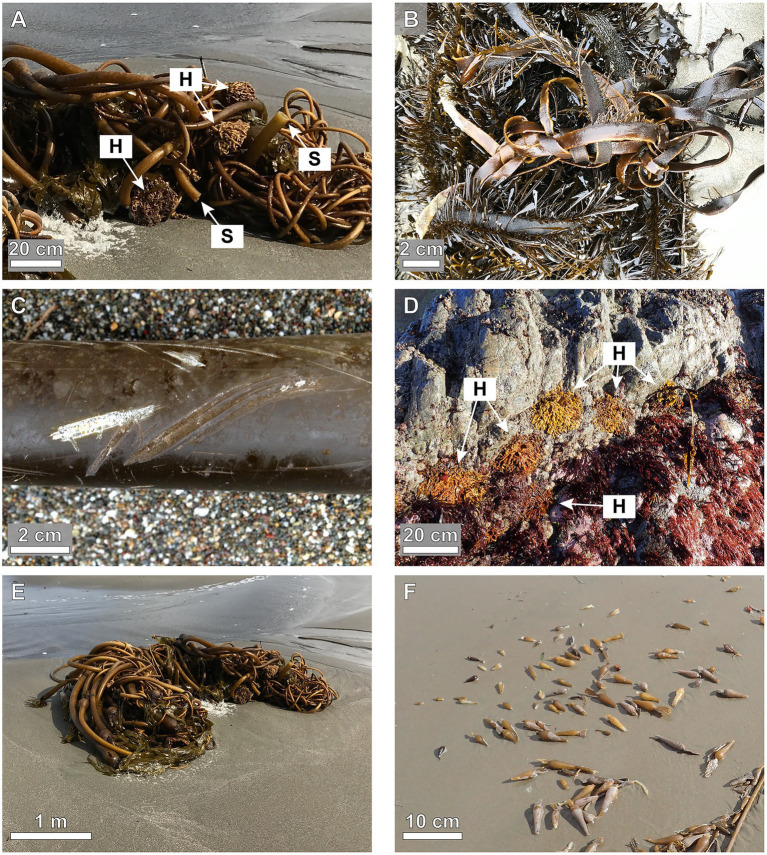
Examples of hydrodynamic damage to macroalgae. **(A)** Kelp, *Nereocystis leutkeana* washed up on the shore, showing broken stipes (S) and detached holdfasts (H). **(B)** Tangled, abraded fronds of kelp, *Egregia menziesii*. **(C)** Abrasions on the stipe of a *N. leutkeana*. **(D)** Holdfasts of *E. menziesii* on a rocky shore after stipes have broken away. **(E)** Beach wrack illustrating the loss of biomass from kelp forests due to hydrodynamic forces on herbivore-damaged *N. leutkeana* (Koehl and Wainwright, 1977). **(F)** Gas-filled floats (pneumatocysts) ripped off at their narrow stems from fronds of *E. menziesii* by moving water and washed ashore.

Moving water damages macroalgae in several ways. Macroalgae are broken if the stress (force per cross-sectional area) imposed by hydrodynamic forces exceeds the strength (stress to break) of their tissues, or are dislodged if stress in the holdfast exceeds attachment strength (Figure 1A; e.g., Koehl, 1986). Moving water can tangle long, flexible algal fronds, increasing hydrodynamic forces and breakage (Figure 1B; Koehl and Wainwright, 1977; Friedland and Denny, 1995; Burnett and Koehl, 2018). Fronds can be abraded as waves scrape them against rough substrata (Figure 1C). Wave-born logs and boulders damage macroalgae as they hit or roll across the shore (Dayton, 1971; Sousa, 1979; Shanks and Wright, 1986), and icebergs scrape away macroalgae (Conlan et al., 1998; Ronowicz et al., 2022).

Consequences of hydrodynamic damage depend on the location of the injury. Dislodgement by holdfast detachment often leads to mortality (Koehl and Wainwright, 1977; Seymour et al., 1989). Stipe breakage removes photosynthetically-active blade tissue (Figure 1D; Santelices et al., 1980; Biedka et al., 1987; Carrington, 1990; Shaughnessy et al., 1996; Bell, 1999; Stewart, 2006b), but does not necessarily cause mortality if drifting thalli survive or if new fronds grow from the holdfast (Lubchenco, 1980; Stewart, 2006b; Loffler et al., 2018; Burnett and Koehl, 2020; Koehl and Daniel, 2022). Biomass loss when blades are damaged is small compared to biomass loss when holdfasts or stipes are broken (Figures 1D,E; Johnson and Mann, 1986; Padilla, 1993; de Bettignies et al., 2013b).

### Herbivores

**Figure 2:**
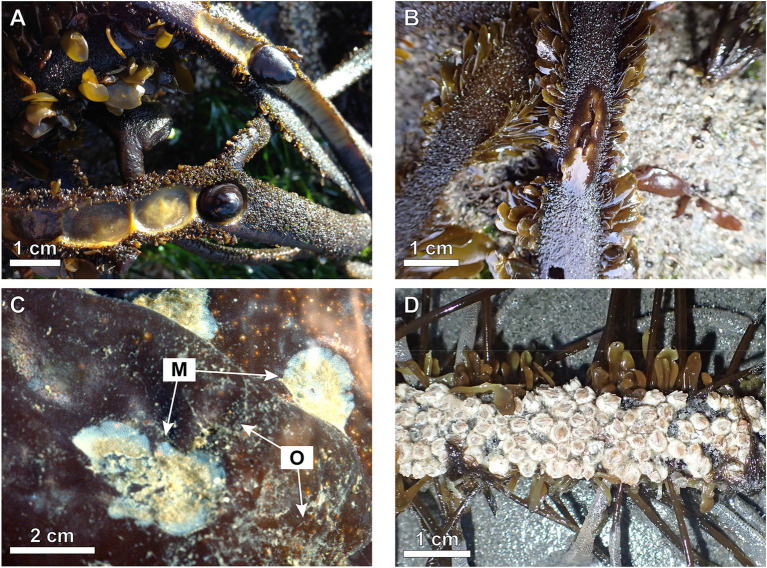
Examples of epibionts on macroalgae. **(A)** Damage of *Egregia menziesii* by herbivorous limpets, *Discurria insessa*. **(B)** Damage of *E. menziesii* by a burrowing, herbivorous amphipod. **(C)** Suspension-feeding encrusting bryozoans, *Membranipora membranacea* (M), and stoloniferous hydroids, *Obelia longissima* (O), growing on the blades of the red alga, *Mazzaella splendens*. **(D)** Barnacles encrusting a frond of *E. menziesii.*

Herbivores, such as limpets and amphipods, damage macroalgae by consuming tissue (Figures 2A,B; Black, 1976; Lowell et al., 1991; de Bettignies et al., 2012). Herbivore bites can lead to further damage by hydrodynamic forces because the cross-sectional area of tissue withstanding those forces is reduced at the bite, so stress is locally higher and can exceed tissue strength (Koehl and Wainwright, 1977; Burnett and Koehl, 2019, 2020). Whether hydrodynamic force on a macroalga causes a crack to propagate across a stipe or blade from a herbivore-inflicted wound depends on the stress-concentration at the crack tip, which is determined by wound shape (Mach et al., 2007; Mach, 2009) sharp cuts inflicted by sea urchins (Koehl and Wainwright, 1977) are more likely to cause breakage than are blunt wounds caused by amphipods and limpets (Black, 1976; Santelices et al., 1980; Gutow et al., 2020). Furthermore, small injuries can enlarge with repeated loading (as in waves), leading to fatigue fracture of a thallus (Mach, 2009). Thus, macroalgal biomass lost due to herbivory is frequently much greater than the tissue consumed by the herbivores (Koehl and Wainwright, 1977; Padilla, 1993).

Population densities of algae-eating animals and the species composition of herbivore communities are affected by local biological interactions (recruitment, competition, predation) and physical factors (water and air temperature, wave height; e.g., Gunnill, 1984; Paine, 1992; Duggins et al., 2001; de Bettignies et al., 2013b; Burnett et al., 2021). Therefore, the degree and nature of herbivore damage to macroalgae varies geographically and seasonally. Generally herbivores damage macroalgae during months when storm activity is low (thus hydrodynamic forces are small), but macroalgae experience increased breakage at herbivore wounds in subsequent months when storms are frequent (Johnson and Koehl, 1994; de Bettignies et al., 2012, 2013b; Burnett and Koehl, 2020).

### Epibionts

Epibionts are organisms living on surfaces of other organisms. Some epibionts on macroalgae are herbivores, but many others do not consume host tissue (see examples in Figures 2C,D; e.g., algae, bryozoans, hydroids, tube worms, barnacles; Koehl and Daniel, 2022). Increased pH under attached epibionts damages host tissues (Wahl, 1989; Harder, 2009), as do anchoring hooks of epibionts (da Gama et al., 2014). Epibionts also damage macroalgae by increasing hydrodynamic forces that their hosts bear by enlarging the structure exposed to ambient flow (Anderson and Martone, 2014), or by stiffening the host, thereby interfering with its reconfiguration by moving water (Koehl and Daniel, 2022).

## Damage prevention

### Reduction of hydrodynamic forces

Flexibility reduces hydrodynamic forces in several ways. Flexible macroalgae in moving water bend over parallel to the flow and reconfigure into streamlined shapes (blades fold into compact forms; branches and blades collapse together into bundles) that reduce wake size and form drag (Koehl, 1984, 1986, 2022; Koehl and Alberte, 1988; Carrington, 1990; Martone et al., 2012; de Bettignies et al., 2013a; Breitkreutz et al., 2022). Furthermore, flexible macroalgae bent close to the substratum encounter slowed flow in the benthic boundary layer (Koehl, 1984; Stewart, 2004, 2006a). However, flexibility sometimes increases drag if fluttering in flow increases wake size (Koehl and Alberte, 1988; Koehl et al., 2008). Species with fleshy blades are better able to reconfigure in flow than are highly branched species (Boller and Carrington, 2007; Starko et al., 2015), and ruffled blades flutter at greater amplitude and experience higher drag than flat blades (Koehl and Alberte, 1988). Many macroalgae are morphologically plastic and grow into drag-reducing shapes in habitats with rapid flow (Koehl et al., 2008). In kelps, this growth response is triggered by tensile stress (Coleman and Martone, 2020; Koehl and Silk, 2021).

In the oscillatory flow at wave-swept habitats, flexible macroalgae move back and forth with the water motion in waves. When moving *with* the flow, water velocities and accelerations *relative to* their surfaces are low, so hydrodynamic forces are small (Koehl, 1984, 1986, 1999, 2022; Burnett and Koehl, 2017). However, when macroalgae reach the end of their tethers, they experience large inertial forces if they were moving rapidly right before being jerked to a halt (Gaylord and Denny, 1997; Denny et al., 1998; Gaylord et al., 2008). Once macroalgae are fully extended, they encounter ambient flow relative to them. Therefore, very long flexible macroalgae in waves may not experience flow past their surfaces or high forces, while shorter macroalgae can reduce hydrodynamic forces if they become fully extended at some point in the wave cycle when water velocities and accelerations are low (Koehl, 1984, 1999, 2022; Wolcott, 2007). Since force on a macroalga in waves depends on its length relative to the distance the water in a wave travels before reversing direction, breakage that shortens a thallus can have profound effects on subsequent damage.

Macroalgae often grow in aggregations (kelp forests, intertidal algal beds). These canopies decrease water speeds, damp wave action, and alter turbulence, so macroalgae in the middle of aggregations experience smaller hydrodynamic forces than isolated macroalgae or those at aggregation edges (Koehl and Alberte, 1988; Johnson, 2001; Gaylord et al., 2007; Zhu et al., 2021a; Koehl, 2022).

### Morphological features and tissue mechanical properties that resist damage

Macroalgae avoid breaking in ambient flow if stresses in their tissues due to hydrodynamic forces are lower than their tissue strength (e.g., Koehl and Wainwright, 1977; Johnson and Koehl, 1994). The distribution of mechanical stresses in macroalgae are calculated using engineering structural analysis (Wainwright et al., 1982), which reveals that macroalgae loaded in tension by ambient flow experience much lower stresses for a given force than do seaweeds bent by the flow, and that wider regions of a thallus experience lower local stresses than do narrow ones (Koehl, 1984, 1999).

Whether local stresses in a macroalga cause damage depends on the mechanical properties of its tissues, which are composite materials composed of cells with fiber-reinforced walls (calcified in some species) and polymeric intercellular matrix (Koehl, 1999; Martone, 2006). Tissue mechanical properties (e.g., strength, extensibility, toughness, resilience) are measured using techniques from materials science (Koehl and Wainwright, 1985). One defense against breakage is having tissues strengthened by calcification or by thick fiber-reinforced cell walls aligned with the directions of highest imposed stresses in the thallus (Padilla, 1993; Koehl, 1999; Martone, 2006; Janot and Martone, 2016; Starko et al., 2018). Another defense against breakage for macroalgae exposed to transient high forces is having very extensible, resilient tissues that do not have time to be stretched to breaking extension during a pulse of force, and that bounce back to their unstretched length before the next pulse (Koehl and Wainwright, 1977). Cells in such tissues are separated by a deformable intercellular matrix, and fibers in their thin walls are oriented at high angles relative to their long axes (Koehl and Wainwright, 1977; Koehl, 1999). Both strategies (strength or extensibility) render a macroalga tough (work/volume to break is high; Koehl, 1999).

Macroalgal tissue strength differs between species (Koehl, 2000; Harder et al., 2006; Krumhansl et al., 2015), within species between habitats (Johnson and Koehl, 1994), and within an individual between support, photosynthetic, and reproductive structures (Demes et al., 2013). Tissue mechanical properties also change with age, growth rate, and season (Johnson and Koehl, 1994; Koehl, 1999; Burnett and Koehl, 2019; Sirison and Burnett, 2020; Koehl and Silk, 2021; Millar et al., 2021). Because flow conditions also vary with time, environmental stress factor (ESF) is used to characterize the resistance of a macroalga to breaking at a defined stage in its life. ESF is the ratio of the season-dependent stress required to break a macroalga to the maximum flow-induced stress it experiences in its habitat during that season (Johnson and Koehl, 1994). Many macroalgae have high ESF’s during the calm summer growth and reproduction season, but low ESF’s during winter, as they accumulate damage and experience storms (Johnson and Koehl, 1994; Koehl and Daniel, 2022). Some species develop similar ESF’s in rapid-flow habitats as in calm sites by increasing tissue strength or cross-sectional area of support structures, and/or by growing into low-drag morphologies (Johnson and Koehl, 1994; Sirison and Burnett, 2020; Koehl and Daniel, 2022).

### Defenses against herbivores and epibionts

Macroalgae use chemical and mechanical defenses against herbivores and epibionts (Padilla, 1989, 1993; Wahl, 1989; Paul, 1992; Steinberg and De Nys, 2002; Walters et al., 2003; Amsler and Fairhead, 2005; da Gama et al., 2014; Koehl and Daniel, 2022). However, epibionts can avoid defended surfaces by preferentially settling in wounds (Black, 1974).

Several hydrodynamic mechanisms remove epibionts from macroalgae. Flowing water can rip epibionts off macroalgae (Fralick et al., 1974; Duggins et al., 2001; Toth and Pavia, 2002; Chenelot and Konar, 2007; Anderson and Martone, 2014). Some macroalgae enhance this removal by shedding their cuticle or surface cell layers (Wahl, 1989; Wahl et al., 1998; Walters et al., 2003; Harder, 2009). As macroalgae with extensible tissues are stretched and bent by ambient flow, stiff animals (e.g., encrusting bryozoans, calcareous tubeworms) crack and pop off their surfaces (Walters et al., 2003; Koehl and Daniel, 2022). Flexible seaweeds flapping in waves can sweep herbivores off the surrounding substratum (Santelices et al., 1980; Kennelly, 1989; Hughes, 2010). When flow breaks off injured parts of macroalgae infested with herbivores, those animals are removed and cannot damage the remaining thallus (Black, 1976; Wahl, 1989, 2008; Wahl and Hay, 1995).

The role of tissue strength and toughness in herbivore deterrence can be determined if mechanical properties of the tissues attacked by herbivores are measured on the spatial scale of herbivore biting or rasping structures (Padilla, 1985). Studies measuring mechanical properties and chemical deterrents showed that tissue toughness is not always a defense against herbivory (Padilla, 1985, 1989; Martone et al., 2021). Similarly, puncture resistance correlates with reduced grazing rates for some macroalgae (Taylor et al., 2002), but not others (Steinberg, 1985).

## Damage management

### Controlling patterns of breakage

Macroalgae can reduce tissue loss *via* structural designs that direct where breakage occurs, and tissue properties that determine how cracks propagate across thalli. For example, some species localize where bending occurs by having joints (narrow regions with flexible tissues; Koehl, 1999; Martone, 2006; Janot and Martone, 2016; Janot et al., 2022). If breakage occurs at such localized regions of high stress, a macroalga can be pruned by ambient flow (Figure 1F) rather than ripped off the shore (Martone, 2006; Martone and Denny, 2008). When cracks propagating through macroalgal tissues are diverted at interfaces between the intercellular matrix and cell walls, more mechanical work is needed to drive the cracks across the structure (Vincent, 2012). Distribution and orientation of fibers and calcification also determines the direction of tears in algae (like rip-stop fabric; Padilla, 1993), for example, causing blades to rip longitudinally without tissue loss.

### Growth and healing in response to damage

After damage occurs, some macroalgae increase the strength of tissue around the wound (Lowell et al., 1991; Toth and Pavia, 2006), while others increase the cross-sectional area of the damaged structure (Burnett and Koehl, 2019). Some damaged macroalgae grow new fronds, becoming bushier (Black, 1974; Fox, 2013). Damage that prunes macroalgae to smaller size reduces their danger of washing away in some cases (Black, 1976; Wolcott, 2007; de Bettignies et al., 2012), but not in others (Burnett and Koehl, 2020). However, excessive damage may leave macroalgae less able to heal or grow (Poore et al., 2018), leading to stunted size or death (Toth and Pavia, 2006; O’Brien and Scheibling, 2016; Pfister and Betcher, 2018; Burnett and Koehl, 2020).

### Life history strategies that compensate for or utilize damage

Some perennial macroalgae persist in rapid-flow habitats by putting their resources into producing strong thalli (thus growing slowly and delaying reproduction), while other species are successful at such sites by growing rapidly and reproducing before seasonally-predictable storms rip their weak thalli off the shore (Santelices et al., 1980; Johnson and Koehl, 1994; Koehl, 1999; Wolcott, 2007). Some macroalgae with “bad” mechanical designs regrow from perennial holdfasts (Bell, 1999), and some recruit opportunistically year-round (Santelices et al., 1980).

Some macroalgae increase the strength of reproductive tissues so they are not prematurely damaged (Demes et al., 2013), whereas others use damage to enhance reproduction and dispersal. For example, *Turbinaria ornata* are weaker and more buoyant when reproductive, so hydrodynamic forces break their stipes and they form floating aggregations where they release gametes and are transported to new sites by currents (Stewart, 2006b). Similarly, drifting in ocean currents by broken-off reproductive kelp aids long-distance dispersal (Bernardes Batista et al., 2018; Fraser et al., 2020, 2022).

## Discussion

Studying the biomechanics of damage to macroalgae from an ecological perspective reveals some surprises. For example, the assumption that an increase in size leads to larger hydrodynamic forces and greater risk of breakage is not necessarily true for macroalgae in waves. Furthermore, while biomechanical analyses show why certain macroalgae have “bad” engineering designs prone to damage, ecological studies reveal how such breakable organisms survive and reproduce in mechanically-stressful environments. Damage can play positive roles in the survival, reproduction, and dispersal of macroalgae. Moreover, damage to macroalgae that outcompete other organisms for space and light can have positive consequences for the local community, enhancing the diversity, growth rates, and abundance of other organisms (Sousa, 1979; Hughes, 2010; Clements et al., 2018).

There are gaps in our current knowledge of macroalgal damage. Little is known about pathways by which cells recognize damage and initiate repairs, whole-thallus signaling that initiates the formation of new fronds when old ones break, or cellular mechanisms that transduce mechanical stresses experienced in nature into patterns of cell division, enlargement, and cell wall construction. Future research should also explore the ecological biomechanics of damage across a greater diversity of macroalgal species, habitats, and life stages to identify ecological patterns and evolutionary histories of mechanisms of damage prevention and management, and to gain insights about the sensitivities of these processes to environmental stress.

## Author contributions

All authors listed have made a substantial, direct, and intellectual contribution to the work, and approved it for publication.

## Funding

NB’s research reviewed here was supported by a National Science Foundation Graduate Research Fellowship DGE-0903711, and MK’s research reviewed here was supported in part National Science Foundation Grant OCE-9217338.

## Conflict of interest

The authors declare that this paper was written in the absence of any commercial or financial relationships that could be construed as a potential conflict of interest.

## Publisher’s note

All claims expressed in this article are solely those of the authors and do not necessarily represent those of their affiliated organizations, or those of the publisher, the editors and the reviewers. Any product that may be evaluated in this article, or claim that may be made by its manufacturer, is not guaranteed or endorsed by the publisher.
